# Corticosteroids in chronic inflammatory demyelinating polyneuropathy: A retrospective, multicentre study, comparing efficacy and safety of daily prednisolone, pulsed dexamethasone, and pulsed intravenous methylprednisolone

**DOI:** 10.1007/s00415-018-8948-y

**Published:** 2018-07-02

**Authors:** G. G. A. van Lieverloo, S. Peric, P. E. Doneddu, F. Gallia, A. Nikolic, L. Wieske, C. Verhamme, I. N. van Schaik, E. Nobile-Orazio, I. Basta, F. Eftimov

**Affiliations:** 1 Academic Medical Centre, University of Amsterdam, Amsterdam, The Netherlands; 2 Neurology Clinic, Clinical Centre of Serbia, School of Medicine, University of Belgrade, Belgrade, Serbia; 3 Neuromuscular and Neuroimmunology Service, Department of Medical Biotechnology and Translational Medicine, Humanitas Clinical and Research Centre, Milan University, Rozzano, Milan, Italy

**Keywords:** CIDP, (Cortico)steroids, Immunosuppressive treatment, Peripheral nerve disorder

## Abstract

**Background:**

Chronic inflammatory demyelinating polyneuropathy (CIDP) can be treated with corticosteroids or intravenous immunoglobulins. Various corticosteroid regimens are currently used in CIDP, but it is unknown whether they are equally efficacious. In this retrospective study, we compared efficacy and safety of three corticosteroid regimens in CIDP patients.

**Methods:**

We included treatment naïve patients that fulfilled the EFNS/PNS criteria for CIDP. Patients were treated with corticosteroids according to the local protocol of three CIDP expertise centres. Corticosteroid regimens consisted of daily oral prednisolone, pulsed oral dexamethasone, or pulsed intravenous methylprednisolone. Outcomes were number of responders to treatment, remission rate of treatment responders, overall probability of 5-year remission, and the occurrence of adverse events.

**Results:**

A total of 125 patients were included. Sixty-seven (54%) patients received daily prednisone or prednisolone, 37 (30%) pulsed dexamethasone, and 21 (17%) pulsed intravenous methylprednisolone. Overall, 60% (95% CI 51–69%) responded to corticosteroids, with no significant difference between the three treatment regimens (*p* = 0.56). From the 75 responders, 61% (95% CI 50–73%) remained in remission, during a median follow-up of 55 months (range 1–197 months). The probability of responders reaching 5-year remission was 55% (95% Cl 44–70%), with no difference between the three groups. Adverse events leading to a change in treatment occurred in ten patients (8%). Two patients had a serious adverse event.

**Conclusion:**

Corticosteroids lead to improvement in 60% of patients and to remission in 61% of treatment responders. There were no differences between treatment modalities in terms of efficacy and safety.

## Introduction

Chronic inflammatory demyelinating polyneuropathy (CIDP) is an immune-mediated disease of the peripheral nerves that causes sensory and motor impairment. Approximately 80% of patients respond well to corticosteroids, intravenous immunoglobulin (IVIg), or plasma exchange [1–3]. The decision which treatment to start first in an individual patient is difficult as there are no good predictors of treatment response.

Both corticosteroids and IVIg have their advantages and drawbacks. Corticosteroids are easy to administer, cheap, and may lead to long-term remission in CIDP more often compared to IVIg [4–7].

However, there are concerns with regard to safety during long-term treatment with corticosteroids. Studies suggest that corticosteroids given in pulses during a relatively short period of time have lower rates of serious side effects than long-term daily oral use of corticosteroids [8–10]. To avoid adverse events associated with corticosteroids, most patients in high-income countries are treated with IVIg, which is associated with less adverse events. IVIg has a fast mode of action, but has to be administrated by regular infusions [11]. IVIg is expensive and, therefore, not widely available.

Various corticosteroid regimens are currently used in CIDP and it is unknown whether one regimen is superior over others. Response and remission rates of corticosteroids in CIDP have so far been described in studies with small number of patients or without comparison between treatment regimens [4, 5, 7, 12]. In this retrospective study, we reviewed the response and remission rates, and the occurrence of adverse events of three different corticosteroid treatment protocols.

## Materials and methods

### Study design and patients

Data were collected retrospectively from treatment naive CIDP patients from year 2000 onwards, in three large CIDP centres in Serbia, The Netherlands, and Italy using a predefined questionnaire. For screening of patients, we used only the periods in which corticosteroids were considered the first-line treatment according to local protocols. Patients needed to fulfil the definite, probable, or possible EFNS/PNS criteria for CIDP [10]. Data were collected anonymously from CIDP databases and hospital charts. Approval by the ethics committee was not acquired under applicable national legislation.

### Treatment protocols

In all three centres, corticosteroids were considered the first-line treatment. Patients with severe disability, contraindication for corticosteroids, or pure motor phenotype were treated with IVIg. In Italy, IVIg was also the preferred treatment in patients with fast progressive disease. There was no predefined cut of ‘severe disability’ of ‘fast progression’. In Serbia, IVIg could only be administered with special approval of the Serbian Health Fund. In The Netherlands, treatment protocol was changed in 2013 from dexamethasone to the combination of IVIg and methylprednisolone as the first-line treatment. Dutch patients who were treated after 2013 were, therefore, not screened for this study. Included patients were treated according to the following protocols:

1. Daily prednisone or prednisolone, starting with 1–1.5 mg/kg body weight during the first 6 weeks, tapering to zero during a period of at least 8 months, at discretion of the treating neurologist (Serbia and The Netherlands). Patients in The Netherlands who did not improve were switched to IVIg treatment, while different treatment modalities were used as rescue treatment in Serbia, dependent on availability. As prednisone is metabolized immediately to prednisolone, we will use the term prednisolone throughout for both formulations.
2. Oral pulsed dexamethasone 40 mg per day for 4 days consecutively each month, during 6 months (The Netherlands). In case of insufficient improvement, patient was switched to IVIg treatment.
3. Intravenous pulsed methylprednisolone, starting with 500 mg daily for 4 days (Italy). Patients who showed improvement after the first course of methylprednisolone were treated with at least two additional courses (with 1 or 2 g/month dependent on disease severity). Patients who did not improve were switched to IVIg treatment.
  All patients received osteoporosis prophylaxes, consisting of oral calcium and vitamin D, during corticosteroid treatment.

### Assessment and outcome

Disease severity at baseline was assessed by motor strength and the ability to walk with or without aid. Motor strength was assessed with the Medical Research Council (MRC) sum score (range 0–60; including shoulder abduction, elbow flexion, wrist extension, hip flexion, knee extension, and ankle dorsiflexion) [13]. A dichotomized value of the modified ranking scale (mRS) was used to classify patients who were able to walk without aid (mRS ≤ 3) or needed assistance while walking (mRS ≥ 4) [14].

The primary outcome was the number of responders. Responders were defined as patients who showed any improvement on motor or sensory impairment as captured by the treating neurologist and/or Rankin scale, and who did not require additional treatment for CIDP. All patients who switched to an alternative treatment, due to insufficient response, or discontinued treatment prematurely due to adverse events, were considered non-responders. The primary outcome was assessed 6 months after start of treatment.

The secondary outcome was the remission rate in treatment responders, categorized according to the CIDP disease activity status (CDAS) [15]. Remission (CDAS 1 and 2) was defined as a stable or improving neurological condition, without the need of ongoing treatment. A relapse was defined as any deterioration warranting new treatment. Other outcomes included the probability to reach 5-year remission in responders, time to relapse, and adverse events. Charts were screened for serious adverse events (SAE) and adverse events (AE). AE were considered mild if they did not lead to a change in dose or interval and moderate if they led to a change in dose or interval during treatment period. SAE was defined as an AE that led to discontinuation of treatment, permanent damage, and life-threatening complications or death.

### Statistical analysis

Patient characteristics including gender, age, disease severity and CIDP variants, were compared between treatment groups. Overall analyses were performed by Fisher–Freeman–Halton *t* test, one-way ANOVA, two-tailed *t* test, or Kurskal–Wallis tests, where applicable. Post hoc analyses were performed on baseline variables using a Fisher–Freeman–Halton *t* test or Mann–Whitney *U* test where applicable.

Response rate and remission rate in treatment responders were compared between treatment groups using a Fisher–Freeman–Halton *t* test. The probability to reach a 5-year remission (CDAS 1) after discontinuation of treatment was assessed using Kaplan–Meier curves in the treatment responders and in the total cohort. Relapsing patients and non-responders were scored as an event. Patients with a follow-up duration shorter than 5 years were censored. Between-treatment group comparisons were performed using the log-rank test. Safety was analysed using descriptive statistics. A *p* value of < 0.05 was considered statistically significant. Analyses were performed using SPSS software.

## Results

**Fig. 1:**
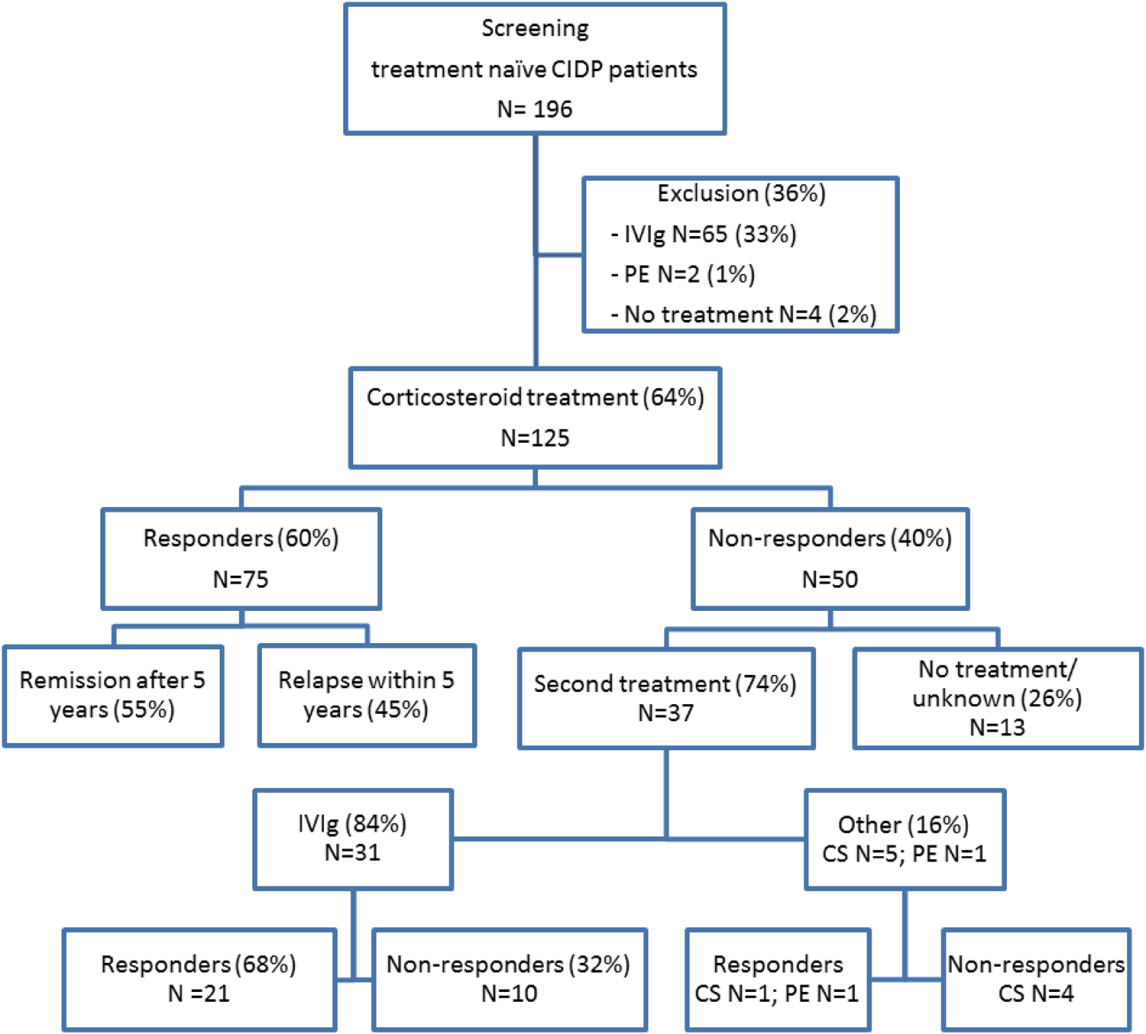
Screening of treatment naïve CIDP patients and treatment response in patients initially treated with corticosteroids. *CS* corticosteroids, *PE* plasma exchange

A total of 196 patients were screened. Of the 67 patients screened in Serbia 58 (87%) were included in the study, five (7%) received IVIg, two (3%) received plasmapheresis, and two (3%) remained untreated. In The Netherlands, 53 CIDP patients were screened; 43 (81%) were included in the study, 8 (15%) received IVIg, and 2 (4%) remained untreated. In Italy, 76 treatment naïve CIDP patients were screened; 24 (32%) were included in the study; 52 (68%) received IVIg (Fig. 1).

We included 125 patients; 98 (78%) had typical CIDP, while multifocal acquired demyelinating sensory and motor neuropathy (MADSAM) was the most common atypical variant (Table 1). Nine patients included in the dexamethasone group and four patients included in the prednisolone group were previously described in the PREDICT trial and were prospectively followed up [4, 10].

**Table 1:** Patients characteristics at baseline per treatment group

	Prednisolone	Dexamethasone	Methylprednisolone	Total	*p* value
Inclusion, *N* (%)	67 (54%)	37 (30%)	21 (17%)	125 (100%)	
The Netherlands	6	37	0	43	
Serbia	57	0	1	58	
Italy	4	0	20	24	
Male, *N* (%)	41 (61%)	29 (78%)	15 (71%)	85 (68%)	0.18
Mean age (SD)	51.1 (18)	55.6 (14)	57.1 (14)	53.4 (16)	0.20
Walking unassisted, *N* (%)	47 (70%)	33 (89%)	20 (95.5%)	100 (80%)	0.01^a^
Median MRC sum score (range)	50 (34–60)	56 (46–60)	57 (42–60)	53 (34–60)	0.003^a^
CIDP subtype, *N* (%)	Typical: 58 (87%) Atypical: 9 (13%) (MADSAM 3, DADS 2, pure motor 4)	Typical: 28 (76%) Atypical: 9 (24%) (MADSAM 5, pure sensory 3, pure motor 1)	Typical: 12 (57%) Atypical: 9 (43%) (MADSAM 4, DADS 2, pure sensory 3)	Typical: 98 (78%) Atypical: 27 (22%) (MADSAM 12, DADS 4, pure sensory 6, pure motor 5)	0.03^a^

*MRC* Medical Research Council sum score (six paired muscle groups), *MADSAM* multifocal acquired demyelinating sensory and motor neuropathy, *DADS* distal-acquired demyelinating symmetric polyneuropathy

^a^Post hoc analysis

Sixty-seven (54%) patients were treated with daily oral prednisolone, 37 (30%) with pulsed oral dexamethasone, and 21 (17%) with iv pulsed methylprednisolone (Table 1). All patients were treated according to protocol, except for three patients. In one patient, prednisolone treatment was stopped after 2 months because of substantial improvement. One patient who was treated with dexamethasone stopped after 5 months, because of substantial improvement and minor side effects. Another patient from the dexamethasone group had a slow improvement and was, therefore, treated for 12 months instead of 6 months. In treatment responsive patients, median duration of treatment with prednisolone was 15 months (range 2–60), leading to a higher cumulative corticosteroid dose compared to the other treatment regimens (Table 2). The prednisolone group included more severely affected patients, compared to the other two treatment regimens [MRC sum score *p* = 0.003 and walking (un)assisted *p* = 0.01]. CIDP subtypes were unevenly distributed between the groups (*p* = 0.03). Gender and age were similar in all groups (Table 1).

**Table 2:** Response rate per treatment group and median duration of treatment in treatment responders

	Prednisolone *N* = 67	Dexamethasone *N* = 37	MPS *N* = 21	Total *N* = 125	*p* value
Treatment response					
Responder, *N* (%) (95% CI)	38 (57%) (45–69%)	25 (68%) (52–83%)	12 (57%) (34–80%)	75 (60%) (51–69%)	0.56
Median duration of treatment (responders only)					
In months (range)	15 (2–60)	6 (5–12)	6.5 (1–60)	6 (2–60)	
Estimated cumulative dose, converted to prednisolone					
Based on 80 kg bodyweight*	10800 mg^a^	6000 mg	9375 mg		

*MPS* methylprednisolone

^a^Based on 80 kg bodyweight

### Primary outcome

Seventy-five CIDP patients (60%, 95% CI 51–69%) were considered responders after corticosteroid treatment; 57% after prednisolone, 68% after dexamethasone, and 57% after methylprednisolone treatment (Table 2). There was no significant difference in response rate between the three treatment regimens (*p* = 0.56). Response to steroids was seen in 3 of 12 (25%) patients with multifocal CIDP (MADSAM).

### Secondary outcomes

**Fig. 2:**
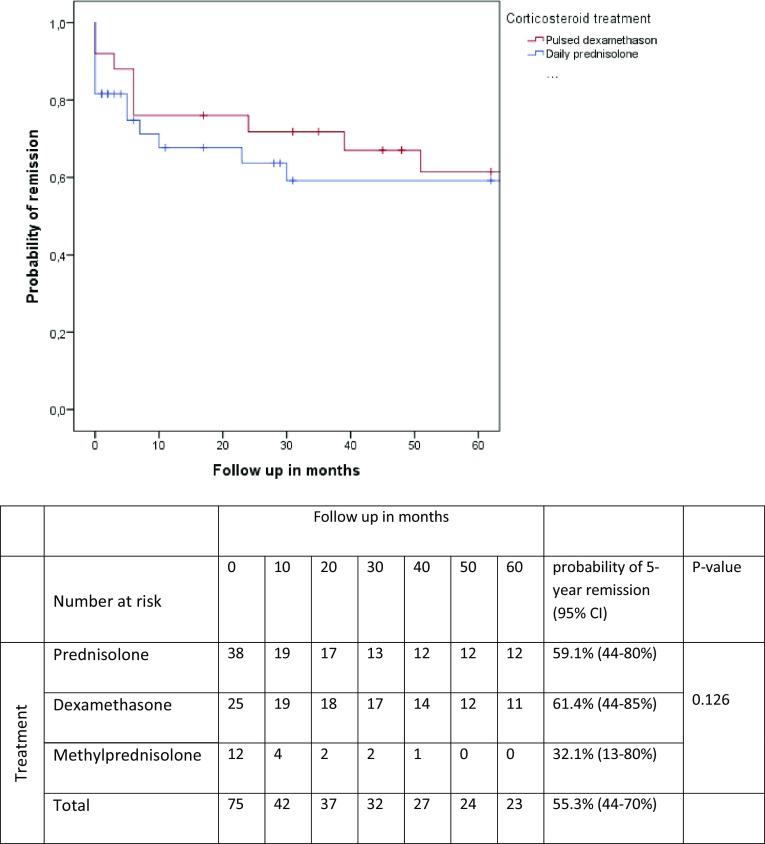
Probability of remission after corticosteroid discontinuation in treatment responders. Data were censored for shorter follow-up duration than 60 months. The MPS group was analysed but removed from figure as the curve was misleading due to the small proportion of responders and large effect of censoring due to short follow-up in most patients

Forty-six of the 75 responders (61%, 95% CI 50–73%) remained in remission during a median follow-up of 55 months (range 1–197, Table 3). Twenty of twenty-nine patients (69%) who experienced a relapse, did so in the first 6 months after treatment withdrawal (Table 3). The probability to reach a 5-year remission (CDAS 1) was 55% (95% CI 44–70%) in patients who responded to corticosteroid treatment (*N* = 75), with no significant difference between the treatment regimens (Fig. 2). Converted to the total cohort (*N* = 125), the probability to reach a 5-year remission after corticosteroids treatment was 33% (Figure not shown). Thirty-one of the fifty non-responders were treated with IVIg of whom 21 (68%) showed a favourable response to IVIg (Fig. 1). Response rate improved to 91% (96/106), when IVIg was given as a subsequent treatment in case corticosteroid treatment was insufficient. Eleven non-responders did not receive an alternative treatment due to unavailability of IVIg or plasma exchange, five patients received corticosteroids in a different regimen, one patient received plasma exchange, and two patients were lost to follow up (Fig. 1). Eight of the nine MADSAM non-responders had a favourable response to IVIg.

**Table 3:** Proportion of patients in remission and median time to relapses in treatment responders

Treatment responders	Prednisolone *N* = 38	Dexamethasone *N* = 25	MPS *N* = 12	Total *N* = 75	*p* value
Patients in remission, *N* (%) (95% CI)	25 (66%) (50–82%)	16 (64%) (44–84%)	5 (42%) (9–74%)	46 (61%) (50–73%)	0.343
Median follow-up of patients reaching remission (range in months)	31 (1–180)	93 (17–197)	19 (2–45)	55 (1–197)	
Median time to relapse (range in months)	0 (0–30)	6 (0–51)	0 (0–12)	4 (0–51)	
< 6 months, *N*	9	6	5	20	
> 6 months, *N* (range in months)	4 (7–30)	3 (24–51)	2 (8–12)	9 (7–51)	

*MPS* methylprednisolone

### Adverse events

Moderate AE were reported in ten patients (8%); nine patients from the prednisolone group and one from the dexamethasone group. Adverse events included hypertension, diabetes mellitus de novo, glaucoma, depression, cushingoid appearance, and gastro-intestinal complaints. SAE occurred in two patients, both in the prednisolone group: one case of severe hypertension and one acute myocardial infarction.

## Discussion

### Response

The three different corticosteroid regimens had a similar response rate of approximately 60%. Reported response rates of various corticosteroid treatment regimens vary between 50 and 90%, which is in line with our results [7–10, 12, 16–18]. A large Italian retrospective study found a similar response rate of 64% after prednisolone treatment, which was not significantly lower than the response rate of IVIg (78%) [12]. We found a relatively poor response to corticosteroids in patients with MADSAM (25%) compared to the literature [19].

### Remission

In this study, we found a remission rate of 61% (46/75) in the treatment responders, with a median follow-up of 55 months (range 1–197). Importantly, two-third of patients who relapsed, did so in the first 6 months after treatment withdrawal. It might be reassuring to patients that the chance of relapse decreases over time.

The probability to reach a 5-year remission (CDAS 1) was 55% (95% CI 44–70%) in patients who responded to corticosteroid treatment (*N* = 75) and 33% in the total cohort (*N* = 125). There were no significant differences between the three corticosteroid regimens in the remission rate and the probability of 5-year remission.

Remission rates found in our study are higher than the long-term remission rates previously reported in smaller studies. During a median follow-up of 42 months in the IMC trial, remission rates were 15% (2/13) after intravenous methylprednisolone and 4% (1/28) after IVIg [5]. However, not all patients in this trial were treatment naïve, which might have led to selection bias to patients with more chronic disease course. The prospective extension study of the PREDICT trial, which included only treatment naïve patients, showed a remission rate of 25% (6/24) in pulsed dexamethasone and 12.5% (2/16) in prednisolone, after a mean follow-up period of 4.5 year [4].

Evidence about long-term remission after withdrawal of IVIg treatment is also limited. In the extension phase of the ICE trial, 57 CIDP patients who responded to IVIg treatment were re-randomized to continue IVIg treatment or to switch to placebo. After 24 weeks of follow-up, 55% of the patients treated with placebo were still in remission [20]. Two retrospective studies provide remission data in patients treated with IVIg. Querol et al. described a cohort of 86 CIDP patients treated with IVIg treatment. After a mean follow-up of 3.9 years, 26% was in remission, 65% was still in need of maintenance treatment and 9% were non-responsive to IVIg [21]. Kuitwaard et al. found a remission rate of 40% (86/214) in CIDP patients responsive to IVIg treatment, with a mean follow-up duration of 5.2 years [22]. The remission rate found in our study is higher and supports the hypothesis that corticosteroids can lead to long-term remission in CIDP more often compared to IVIg. However, comparison between these retrospective studies with possible selection bias should be performed with caution. In addition, chance of remission might also be related to certain clinical features, such as symmetrical distribution or a relapsing–remitting course [6].

### IVIg in non-responders

When corticosteroid therapy failed and IVIg was given as a subsequent treatment, response rate improved to 91% (96/106). Cocito et al. found that 86% (108/125) of patients had a good response to corticosteroids, or to subsequent IVIg treatment, in case the initial corticosteroids treatment failed [12]. Alternatively, Kuitwaard et al. studied CIDP patients initially treated with IVIg [22]. They found a similar response rate of 94% (234/248) in patients who had a good response to the initial IVIg treatment, or who received corticosteroids as subsequent treatment when IVIg failed. Both corticosteroids and IVIg treatment are considered the first-line treatment. In general, IVIg is regarded to have slightly higher response rates than corticosteroids [12, 16, 22]. However, the results from these studies suggest that most patients respond to at least one of both treatments, and that the overall response rate does not depend on the sequence of treatments.

### Adverse events

Moderate AE occurred in 8% of patients of whom two patients had an SAE. Most of these adverse events were seen in the prednisolone group, which was the largest group of the three treatment regimens. Another possible explanation for the number of adverse events in the prednisolone group was the higher cumulative dose of prednisolone. The number of mild AE, which did not lead to a change in dose or interval, was low. Given the retrospective nature of this study, we suspect that mild AE were underreported and, therefore, not representative for the true number of mild AE occurring during corticosteroid treatment.

A known and serious side effect from long-term use of corticosteroids is osteoporosis. The American College of Rheumatology Guideline for the Prevention and Treatment of Glucocorticoid-Induced Osteoporosis recommends to treat all patients with calcium and vitamin D when starting long-term corticosteroid treatment [23]. Patients in this study were all treated accordingly. No osteoporotic fractures occurred in our cohort, but DEXA scans were not performed routinely to check for the occurrence of osteoporosis.

### Strength and limitations of our study

The main limitation of this study is the retrospective nature and lack of standardized evaluation and follow-up of patients. Moreover, there were no predefined selection criteria for the first-line treatment other than severe disability and contraindication for corticosteroids that can be interpreted in different ways. This could have led to selection bias. In Italy, for example, a majority of patients were treated with IVIg in contrast with Serbia and the Netherlands. Although ‘fast progression’ was used as an additional criterion for use of IVIg, it is unlikely that this criterion explains the large differences between the percentages of patients treated with IVIg in the three centres. In Serbia, IVIg is only available with special approval of the Serbian Health Fund; therefore, most patients were treated with prednisolone. This might explain why the prednisolone group contained more severely impaired patients. It is, however, unclear whether severity of disability is a determinant of treatment response or remission [24]. As the treatment regimens were largely centre specific, other confounders could have attributed to the reported response and remission rates, including health care infrastructure and the availability of physiotherapy and rehabilitation.

Another possible limitation is the difference in treatment duration between the different corticosteroid regimens. The median treatment duration of the prednisolone group was 15 months, leading to a higher cumulative dose compared to the dexamethasone and methylprednisolone groups. Alternatively, in the methylprednisolone group, the treating neurologist decided after a single course of methylprednisolone whether to continue methylprednisolone or to switch to IVIg, based on clinical improvement of the patient. The decision whether dexamethasone or prednisolone treatment was effective or not was made after several months of treatment. Previous studies have shown that time to improve after corticosteroids can take up to several months. Measuring outcome after four weeks in the methylprednisolone group might have been too early for some patients and might have caused an underestimation of the response rate [8, 10]. Despite the limitations of a retrospective study, we believe that these results provide insight in everyday CIDP practice and will be helpful for treating neurologists, in both high-income and low-income countries. Only a few comparative studies are available regarding corticosteroid treatment in CIDP and new randomized trails are not likely to be undertaken. To our knowledge, this is the largest study comparing different corticosteroids regimens in treatment naïve CIDP patients.

## Conclusions

We conclude that the response rate of corticosteroids as the first-line treatment for CIDP is 60%, without a significant difference between the three regimens. About a third of all patients will remain in remission after treatment with corticosteroids. Most patients who experience a relapse do so in the first 6 months after discontinuation of treatment. Although there were no differences in response and remission rate between the regimens, pulsed corticosteroids regimens have lower cumulative doses and possibly less long-term adverse events. A treatment protocol with corticosteroids, followed by IVIg as a subsequent treatment in case corticosteroid treatment is insufficient, can lead to improvement in 9 out of 10 patients.
